# Lipidized prolactin-releasing peptide improved glucose tolerance in metabolic syndrome: Koletsky and spontaneously hypertensive rat study

**DOI:** 10.1038/s41387-017-0015-8

**Published:** 2018-01-16

**Authors:** Barbora Mikulášková, Martina Holubová, Veronika Pražienková, Jana Zemenová, Lucie Hrubá, Martin Haluzík, Blanka Železná, Jaroslav Kuneš, Lenka Maletínská

**Affiliations:** 1 Institute of Organic Chemistry and Biochemistry AS CR, Prague, Czech Republic; 2 Institute of Physiology AS CR, Prague, Czech Republic; 3 University of Chemistry and Technology, Prague, Czech Republic; 4 Centre for Experimental Medicine and Diabetes Centre, Institute for Clinical and Experimental Medicine, Prague, Czech Republic; 5 Institute of Medical Biochemistry and Laboratory Diagnostics, First Faculty of Medicine, Charles University in Prague and General University Hospital, Prague, Czech Republic

## Abstract

**Background/Objectives:**

Prolactin-releasing peptide (PrRP) has a potential to decrease food intake and ameliorate obesity, but is ineffective after peripheral administration. We have previously shown that our novel lipidized analogs PrRP enhances its stability in the circulation and enables its central effect after peripheral application. The purpose of this study was to explore if sub-chronic administration of novel PrRP analog palmitoylated in position 11 (palm^11^-PrRP31) to Koletsky-spontaneously hypertensive obese rats (SHROB) could lower body weight and glucose intolerance as well as other metabolic parameters.

**Subjects/Methods:**

The SHROB rats (*n* = 16) were used for this study and age-matched hypertensive lean SHR littermates (*n* = 16) served as controls. Palm^11^-PrRP31 was administered intraperitoneally to SHR and SHROB (*n* = 8) at a dose of 5 mg/kg once-daily for 3 weeks. During the dosing period food intake and body weight were monitored. At the end of the experiment the oral glucose tolerance test was performed; plasma and tissue samples were collected. Thereafter, arterial blood pressure was measured.

**Results:**

At the end of the experiment, vehicle-treated SHROB rats showed typical metabolic syndrome parameters, including obesity, glucose intolerance, dyslipidemia, and hypertension. Peripheral treatment with palm^11^-PrRP31 progressively decreased the body weight of SHR rats but not SHROB rats, though glucose tolerance was markedly improved in both strains. Moreover, in SHROB palm^11^-PrRP31 ameliorated the HOMA index, insulin/glucagon ratio, and increased insulin receptor substrate 1 and 2 expression in fat and insulin signaling in the hypothalamus, while it had no effect on blood pressure.

**Conclusions:**

We demonstrated that our new lipidized PrRP analog is capable of improving glucose tolerance in obese SHROB rats after peripheral application, suggesting that its effect on glucose metabolism is independent of leptin signaling and body weight lowering. These data suggest that this analog has the potential to be a compound with both anti-obesity and glucose-lowering properties.

## Introduction

Obesity and its complications have reached epidemic proportions. Thus, finding new, more effective drugs for the treatment of obesity without any side effects is a major challenge. Natural anorexigenic neuropeptides have that potential, but their major disadvantage is their low stability in plasma and inability to cross the blood brain barrier (BBB) after peripheral administration. One strategy for designing peptidic drugs is based on peptide lipidization^1, 2^. This modification leads to increased peptide stability and half-life in the organism and enables its application to the periphery because lipidized peptides are capable of crossing the BBB. Liraglutide, a palmitoylated analog of glucagon-like peptide 1 (GLP-1)^3^; semaglutide, an analog of GLP-1 with a C-18 fatty di-acid chain^4^; or the insulin analog detemir, which has a myristic acid attached through an amide bond, are examples of lipidized peptide drugs^5^.

We have previously demonstrated that lipidization of prolactin-releasing peptide (PrRP) enhances its stability in the circulation and enables its central effect after peripheral application^6^. We have tested the lipidization of both natural PrRP peptidic forms containing 31 (PrRP31) or 20 (PrRP20) amino acids and sharing identical C-terminal parts using several fatty acids with different lengths. The most significant inhibition on food intake and decrease in body weight (BW) was demonstrated with the use of analogs with palmitic (palm-PrRP31) or myristic (myr-PrRP20) acids attached to the N-terminus^6^. Thus, palm-PrRP31 and myr-PrRP20 were used in our following studies^6–9^. The potential of PrRP for the treatment of obesity was summarized in our recent review^2^. It is evident that 2 weeks of twice-daily administration of palm-PrRP31 and myr-PrRP20 to mice with high-fat diet-induced obesity (DIO) significantly decreased cumulative food intake and BW. The decrease in BW was due to a reduction in the fat mass accompanied by a decrease in circulating leptin levels as well as decreased lipogenesis in adipose tissue^6^. The decrease in food intake and BW as well as improved glucose tolerance was also shown in DIO rats after a 2-week treatment with palm-PrRP31^8^. In contrast, in Zucker diabetic fatty (ZDF) rats, the same treatment with palm-PrRP31 decreased food intake but did not significantly affect BW or glucose tolerance^8^, probably as a consequence of severe leptin resistance due to a nonfunctional leptin receptor^10^. Recently, we have developed a novel PrRP analog palmitoylated at position 11 (palm^11^-PrRP31) with improved bioavailability, demonstrated that it is able to bind to PrRP receptor with high affinity in vitro and shown that this PrRP analog decreased BW and food intake in DIO mice^11^.

The Koletsky rat strain of genetically obese hypertensive rats develops obesity, hyperinsulinemia, hyperlipidemia, and spontaneous hypertension, which are the main symptoms of metabolic syndrome^12, 13^. The Koletsky rats or spontaneously hypertensive obese (SHROB) rats carry the obesity mutation, designated as *fa*^*k*^, a nonsense mutation in the leptin receptor^14^, which means they are incapable of leptin signaling. These rats have 15- to 20-fold greater fasting insulin levels than lean spontaneously hypertensive rats (SHR) and increased insulin secretion in response to oral glucose load. The oral glucose test also demonstrated glucose intolerance, but without overt diabetes. This suggests that the *fa*^*k*^ mutation expressed in SHROB rats is not sufficient to trigger diabetes^15^.

The aim of this study was to explore the effects of 3 weeks administration of palm^11^-PrRP31 to SHROB rats and their SHR controls on food intake and glucose tolerance, as well as relevant metabolic parameters and insulin signaling.

## Materials and methods

### Synthesis of PrRP

Human palmitoylated PrRP31 analog (SRTHRHSMEI K (N-γ-E (N-palm)) TPDINPAWYASRGIRPVGRF-NH_2_) was synthesized and purified as described previously^6^. Palmitoylation at position 11 was performed as shown previously, on a fully protected peptide on a resin as a last step^11^. The purity and identity of the peptide were determined by high-performance liquid chromatography and using a Q-TOF micro MS technique (Waters, Milford, MA, USA).

### Animals and diet

All animal experiments followed the ethical guidelines for work with animals by the Act of the Czech Republic Nr. 246/1992 and were approved by the Committee for Experiments with Laboratory Animals of the Academy of Science of the Czech Republic.

Experiments were conducted on homozygous male SHROB rats (*fa*^*k*^/*fa*^*k*^). Age- and sex-matched hypertensive lean SHR littermates were used as controls for this study. The 6-week-old male rats of both genotypes were purchased from Charles River (Wilmington, USA). Rats were provided food—Ssniff (Spezialdiäten GmbH, Soest, Germany) (58% carbohydrates, 9% fat, 33% protein) and water ad libitum. Animals were on a 12:12-h light–dark cycle (lights on from 5:00 to 17:00 h) and maintained at a constant temperature of 22 ± 2 °C.

### Study design and drug administration

Before the start of treatment, BW was monitored twice a week and fasted blood samples were collected from the tail vessels at 16 weeks for determination of the basic biochemical plasma profile. At the age of 16 weeks, the following four experimental groups were randomly established (*n* = 8): (A) SHR vehicle, (B) SHR palm^11^-PrRP31, (C) SHROB vehicle, and (D) SHROB palm^11^-PrRP31.

palm^11^-PrRP31 was dissolved in 50 mM phosphate-buffered saline, pH = 6 (PBS) (vehicle) for intraperitoneal (IP) administration and applied at a dose of 5 mg/kg once a day (at 15:00 h) in a dosing volume of 1.0 ml/kg IP for 21 days. The dose used in this study was chosen according to previously tested food intake after acute IP administration of palm^11^-PrRP31 in rats (data not shown).

**Fig. 1:**
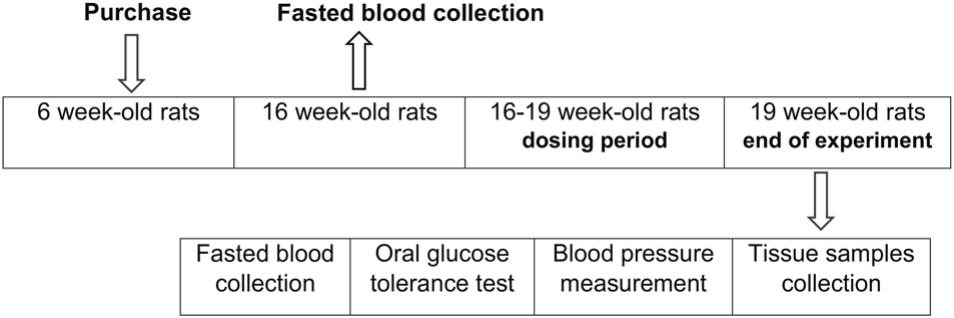
A schematic overview of the study design The SHROB (*n* = 16) and SHR (*n* = 16) were purchased at the age of 6 weeks. Before the start of treatment fasted blood plasma samples were collected from the tail vessels at the age of 16 weeks for determination of the basic biochemical plasma profile. Dosing period started at the age of 16 weeks. palm^11^-PrRP31 was applied intraperitoneally to SHR and SHROB (*n* = 8) at a dose of 5 mg/kg once a day for 21 days. At the end of the experiment rats were overnight fasted, blood samples from the tail vessels were collected for determination of the biochemical plasma parameters, and then the oral glucose tolerance test was performed. Thereafter, arterial blood pressure was measured. The animals were killed by decapitation and tissue samples were collected

BW and food intake were monitored every 2 days during drug application. At the end of the experiment, the rats were fasted overnight, blood samples were collected for determination of the biochemical parameters from the tail vessels, and an oral glucose tolerance test (OGTT) was performed. Thereafter, arterial blood pressure was measured by direct puncture of carotid artery under light ether anesthesia. The animals were killed by decapitation and tissue samples were collected. The liver, fat tissue, kidney, heart, and gastrocnemius muscle were dissected. The tissue samples were weighed, frozen in liquid nitrogen, and stored at −80 °C for further processing. The hypothalami were separated from the dissected brains and homogenized in a Bullet Blender (Next Advance Inc., Averill Park, NY, USA) using lysis buffer^16^ and stored at −20 °C. The experimental design is summarized in Fig. 1.

### Oral glucose tolerance test

Overnight-fasted rats were administered a glucose solution at a dose of 2 g/kg BW by gavage. Blood samples were obtained from the tail vessels of unrestrained, conscious animals into heparinized capillaries at 0, 30, 60, 90, 120, and 180 min. The blood glucose concentrations were determined in whole blood by using the glucose oxidase method (glucose analyzer 8/28 BIOSEN S Line; EKF Diagnostics, Barleben, Germany) and the delta of the area under curve (AUC) was calculated.

### Determination of biochemical parameters

Proteins of interest in the hypothalamus and gastrocnemius muscle were determined using a Pierce BCA protein assay kit (Thermo Fisher scientific Inc, Rockford, IL, USA) and western blotting.

The triglyceride content in the liver was evaluated in chloroform extract using an enzymatic photometric assay (Erba Lachema, Brno, Czech Republic) as described in Papáčková et al.^17^.

Leptin, triglyceride, and insulin plasma levels were determined as described previously^6^. Free fatty acid (FFA) levels were determined by a colorimetric assay (Roche, Mannheim, Germany). The total cholesterol and urea levels in the plasma were measured by enzymatic photometric determination (Erba Lachema) and glucagon plasma levels by RIA assay (Millipore, St. Charles, MI, USA). Cytokines (interleukins (IL) 6, 1β, and 10 and tumor necrosis factor α (TNF-α)) were measured in plasma with a MILIPLEX MAP rat cytokine bead panel (Millipore).

### Western blotting

Hypothalami samples were processed and western blotting was performed as described in ref. ^16^. Muscle samples were processed like the hypothalami samples, only the boiling before electrophoresis was skipped.

The following primary antibodies were used for western blotting: insulin receptor β (IR β), PI3 kinase (PI3K), phospho-mitogen-activated protein kinase (MAPK/ERK1/2), and MAPK/ERK1/2 from Cell Signaling Technology (Beverly, MA, USA) and beta-actin from Sigma Aldrich (St. Louis, MO, USA). The following secondary antibodies were used: anti-mouse IgG HRP-linked antibody and anti-rabbit IgG HRP-linked antibody (Cell Signaling Technology).

### Determination of mRNA

The tissue samples used for mRNA determination were processed as previously described^6^. The mRNA expression of the genes for acetyl-CoA carboxylase 1 (Acaca), glucose transporter type 4 (Glut4), leptin (Lep), lipoprotein lipase (Lpl), peroxisome proliferator-activated receptor (Ppar) γ, stearoyl-CoA desaturase-1 (Scd1), insulin receptor substrate 1,2 (Irs1,2), and sterol regulatory element-binding protein 1 (Srebf1) in white adipose tissue; Glut4 and uncoupling protein 1 (UCP-1) in brown adipose tissue; and Acaca, fatty acid synthase (Fasn), Pparα and Pparγ in liver was examined using an ABI PRISM 7500 instrument (Applied Biosystems, Foster City, CA, USA). The expression of beta-2-microglobulin (B2m) was used to compensate for variations in input RNA amounts and the efficiency of reverse transcription.

### Drug exposure

Blood plasma concentrations of palm^11^-PrRP31 were determined with a rat PrRP(1–31) EIA high-sensitivity kit (Peninsula Laboratories, San Carlos, CA, USA) according to the manufacturer’s instructions. Additional external calibration was completed with rat plasma matrix. The 21-day IP treatment with palm^11^-PrRP31 led to a significant increase in the peptide in the plasma from both treated groups, resulting in peptide plasma levels in the range from 20 to 50 ng/ml.

### Statistics

The results are expressed as the means ± S.E.M. Data were evaluated by unpaired *t*-test or two-way analysis of variance (ANOVA) followed by the Bonferroni post hoc test as indicated in the tables and figures using the GraphPad software (Graph-Pad Software, San Diego, CA, USA). *P* < 0.05 was considered statistically significant. The rate of insulin resistance was expressed with a homeostatic model assessment (HOMA) index calculated as the (fasting glucose level, mmol/l)×(fasting insulin level, pmol/l) divided by 22.5 (ref. ^18^).

## Results

### Characterization of SHROB and SHR rats

The BW of the rats increased during the first 16 weeks (as they aged) (before drug administration) in both genotypes; however, it reached significantly higher values in the SHROB rats compared with the SHR rats (476 ± 5 vs 347 ± 6 g, *P* < 0.001, Table 1). The higher BW in the SHROB rats was accompanied by significantly higher plasma triglycerides, total cholesterol, insulin, and leptin levels as well as HOMA index (Table 1). However, there was no significant difference in fasting blood glucose levels between the strains.

**Table 1:** Metabolic parameters analyzed in fasted blood plasma, organs weights, triglycerides in liver, and systolic blood pressure of SHR and SHROB before treatment (16 weeks of age) and after treatment (19 weeks of age) with palm^11^-PrRP31

Genotype treatment	SHR	SHROB	SHR vehicle	SHR palm^11^PrRP31	SHROB vehicle	SHROB palm^11^PrRP31
Age (weeks)	16		19			
Body weight (g)	347 ± 6	476 ± 5***	373 ± 8	325 ± 10^**#**^	516 ± 8***	508 ± 8
Glucose (mmol/l)	5.08 ± 1.13	5.31 ± 0.12	3.88 ± 0.20	4.12 ± 0.08	4.44 ± 0.59	4.02 ± 0.20
Triglycerides (mg/ml)	0.49 ± 0.04	2.00 ± 0,54***	0.40 ± 0,06	0.38 ± 0.04	1.48 ± 0.17***	2.44 ± 0.88
Cholesterol (mmol/l)	1.90 ± 0.22	2.81 ± 0.32*	1.99 ± 0.38	1.82 ± 0.24	2.40 ± 0.23	3.17 ± 0.55
Free fatty acids (mmol/l)	NT	NT	0.82 ± 0.04	0.97 ± 0.05^#^	0.60 ± 0.07*	0.80 ± 0.05^#^
Leptin (ng/ml)	4.71 ± 0.43	181.70 ± 5.75***	3.97 ± 0.46	2.37 ± 0.39^#^	179.10 ± 8.78***	166.20 ± 17.76
Insulin (ng/ml)	0.47 ± 0.05	22.49 ± 1.03***	0.72 ± 0.08	0.60 ± 0.06	19.73 ± 2.16***	13.08 ± 1.71^#^
HOMA index	18.88 ± 1.87	865.6 ± 45.90***	21.89 ± 3.17	19.32 ± 2.26	734.70 ± 172.20**	411.80 ± 74.37
Glucagon (ng/ml)	NT	NT	34.01 ± 2.12	29.71 ± 2.63	25.65 ± 2.70*	36.32 ± 1.42^###^
Insulin/glucagon ratio	NT	NT	2.20 ± 0.29	2.11 ± 0.26	85.16 ± 14.25***	35.53 ± 3.39^##^
Liver (g)	NT	NT	9.30 ± 0.61	7.90 ± 0.38	19.30 ± 1.01***	19.05 ± 0.96
TAG in liver (mmol/g of protein)	NT	NT	1.23 ± 0.18	0.47 ± 0.08^##^	7.02 ± 0.64***	4.47 ± 1.30
Heart (g)	NT	NT	1.15 ± 0.04	1.03 ± 0.02^#^	1.06 ± 0.04	1.00 ± 0.03
Kidney (g)	NT	NT	2.20 ± 0.06	1.92 ± 0.06^#^	2.28 ± 0.09	2.12 ± 0.08
Urea (mmol/µl)	NT	NT	6.34 ± 0.55	8.51 ± 1.27	7.51 ± 0.56	6.27 ± 0.47
SBP (mmHg)	NT	NT	201.50 ± 8.74	205.30 ± 4.78	177.40 ± 9.41	184.80 ± 8.47

Data are presented as means ± S.E.M. Statistical analysis was performed by unpaired *t*-test. *SBP* systolic blood pressure, *TAG* triglycerides, *NT* not tested. Significance is ^*^P < 0.05, **P < 0.01, ***P < 0.001 vs the lean control SHR group (*n* = 8), ^#^*P* < 0.05, ^##^*P* < 0.01, ^###^*P* < 0.001 vs the respective vehicle-treated control group (*n* = 8)

**Fig. 2:**
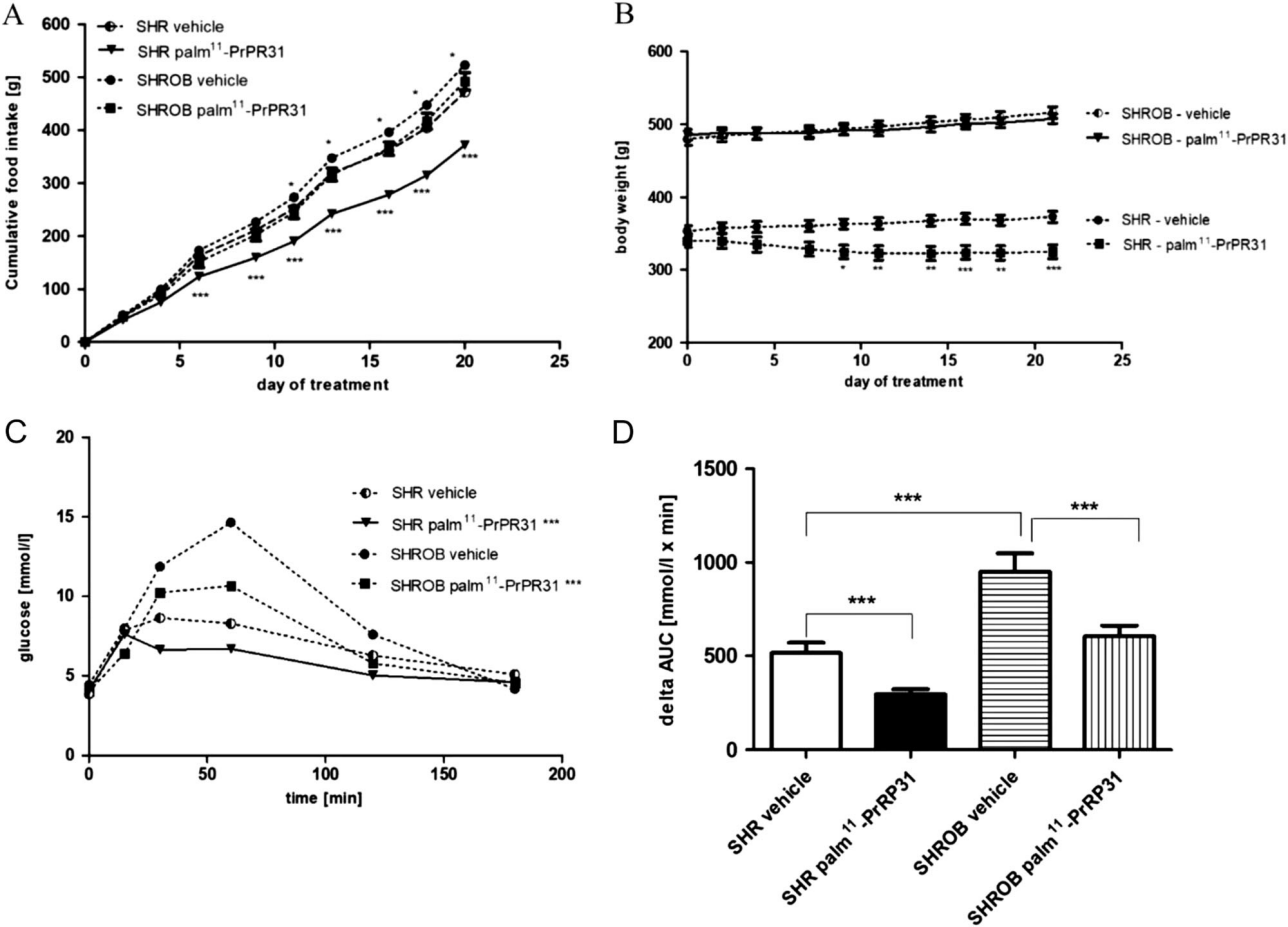
Food intake, body weight and oral glucose tolerance test. Chronic effect of palm^11^-PrRP31 on food intake (**a**), body weight (**b**), and oral glucose tolerance test (OGTT) response (**c, d**) in SHR and SHROB. palm^11^-PrPR31 was administered intraperitoneally at a dose of 5 mg/kg once a day for 21 days. Food intake and body weight were monitored every 2 days during drug application. OGTT was performed at the end of experiment; results are shown as glucose profile and delta AUC. Data are presented as means ± S.E.M. Statistical analysis was performed by repeated measures ANOVA with Bonferroni post hoc test, significance is *P < 0.05, **P < 0.01, ***P < 0.001 vs the vehicle-treated control group (*n* = 8)

Similar differences in the metabolic parameters, as mentioned above, were noted at the age of 19 weeks between the vehicle-treated SHROB and SHR rats (Table 1). Furthermore, higher triglyceride contents in the liver, but lower FFA and glucagon levels and insulin/glucagon ratio were observed. The OGTT revealed impaired glucose tolerance in the SHROB rats compared to the SHR controls (Fig. 2c), which was confirmed by a higher delta AUC (Fig. 2 d).

The liver weights in the SHROB rats were significantly higher than in the SHR rats. The kidney and heart weights did not show differences between the genotypes (Table 1). The systolic blood pressure (SBP) was comparable in both genotypes (Table 1), though it tended to be higher in the SHR rats.

There were no significant differences in measured cytokines (IL-10, TNF-α, IL-6, and IL-1β) in the blood plasma from the SHR and SHROB rats (Suppl. Figure 1A–D).

**Fig. 3:**
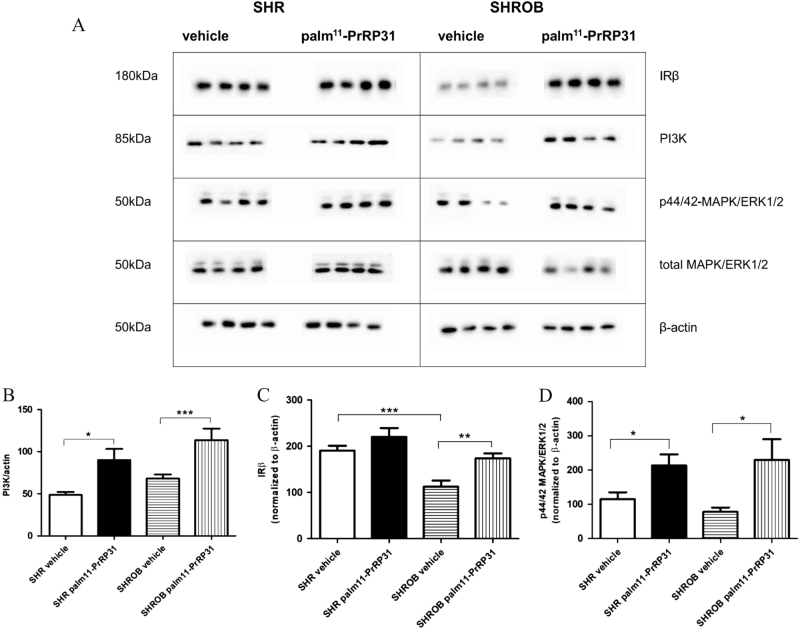
Signaling pathways in hypothalamus Western blots—levels of IRβ, PI3K, and MAPK/ERK1/2 (**a**). Densitometric quantification of western blots normalized to β-actin—PI3K (**b**), IRβ (**c**), and MAPK/ERK1/2/total MAPK/ERK1/2 (**d**). Data are presented as means ± S.E.M. Statistical analysis was performed by unpaired *t*-test. Significance is *P < 0.05, **P < 0.01, ***P < 0.001, vs respective control group (*n* = 8)

At the end of the experiment, significantly lower hypothalamic IRβ was detected in the vehicle-treated SHROB rats compared to the vehicle-treated SHR rats. However, PI3K and MAPK/ERK1/2 phosphorylation of p44/42 were not different between the genotypes (Fig. 3a–d).

SCAT and liver mRNA expressions of Acaca, the rate-limiting step in fatty acid synthesis, as well as SCAT Scd1 and liver Fasn expression were significantly higher in the SHROB than the SHR rats. In contrast, Lpl mRNA expression in SCAT was higher in the SHROB than SHR rats (Table 2 and Suppl. Figure 2A). Moreover, PPARγ mRNA expression was significantly higher in the SHROB rats than the SHR rats in both the SCAT and liver; similar results were also observed for PPARα mRNA expression in the liver. However, in the IPAT, Irs2 and Srebf1 mRNA expression were lower in SHROB rats than in SHR rats. Leptin mRNA expression was higher in the SHROB compared with SHR rats in the SCAT, but did not differ in the IPAT.

**Table 2:** Summary of gene expression in adipose tissue and liver in SHR and SHROB after treatment with palm^11^-PrRP31

Tissue	Gene	SHROB vs SHR	SHR palm^11^-PrRP31 vs vehicle	SHROB palm^11^-PrRP31 vs vehicle
SCAT	*Acaca*	↑***	–	↓
	*Glut4*	↑**	–	–
	*Lep*	↑***	↓*	↓
	*Lpl*	↑*	↓	↓
	*Pparγ*	↑**	↑	↓
	*Scd1*	↑***	↓	↓
IPAT	*Irs1*	–	↑*	↑***
	*Irs2*	↓**	↑	↑***
	*Srebf1*	↓*	↑**	↑*
BAT	*Glut4*	↑***	↑*	↑
	*UCP-1*	↓***	↑	–
Liver	*Acaca*	↑*	↑	↓
	*Fasn*	↑**	–	↓
	*Pparα*	↑**	↓	↓*
	*Pparγ*	↑***	↑	↓

Statistical analysis was performed by unpaired *t*-test

*SCAT* subcutaneous adipose tissue, *IPAT* intraperitoneal adipose tissue, *BAT* brown adipose tissue, *Acaca* acetyl-CoA carboxylase 1, *Glut4* glucose transporter type 4, *Lep* leptin, *Lpl* lipoprotein lipase, *Pparγ* peroxisome proliferator-activated receptor γ, *Scd1* stearoyl-CoA desaturase-1, *Irs1,2* insulin receptor substrate 1,2, *Srebf1* sterol regulatory element-binding protein 1, *UCP-1* uncoupling protein 1, *Fasn* fatty acid synthase, ***↓*** decrease, *↑* increase Significance is *P  <  0.05, **P  <  0.01, ***P  <  0.001 vs the respective vehicle-treated control group (*n * =  8). The expression of particular genes was normalized to beta-2-microglobulin (B2m)

In the BAT, UCP-1 mRNA expression was negligible in the SHROB rats. The expression of Glut4 was significantly higher in the SHROB compared to SHR rats in both the BAT and SCAT (Table 2 and Suppl. Figure 2).

### The effect of palm^11^-PrRP31 on food intake, BW, biochemical and metabolic parameters, and signaling in the hypothalamus

Treatment with palm^11^-PrRP31 lowered food intake in both treated groups, though the effect was more pronounced in the SHR rats compared to SHROB rats (Fig. 2a). Similarly, BW was reduced significantly in the SHR rats (−13%, *P* < 0.001), while BW changes were minimal in the SHROB rats after palm^11^-PrRP31 treatment (Fig. 2b).

Fasting plasma glucose levels in the SHR and SHROB rats were not affected by the treatment. However, palm^11^-PrRP31 administration improved tolerance to glucose via the OGTT in both genotypes (Fig. 2c, d) according to the significantly decreased AUC in the palm^11^-PrRP31-treated groups compared to the vehicle-treated groups.

palm^11^-PrRP31 administration did not affect fasting plasma triglycerides or cholesterol levels either the SHR or SHROB rats. In the SHROB rat model, palm^11^-PrRP31 treatment insignificantly decreased leptin plasma levels (Table 1) and significantly increased FFA plasma levels. In the SHR rats, the treatment significantly decreased leptin and increased FFA in the plasma (Table 1). Liver triglycerides were significantly decreased in the SHR rats after the treatment (Table 1).

Increased plasma glucagon levels but lowered insulin levels resulting in a decreased insulin/glucagon ratio were observed in the SHROB rats after treatment. In contrast, no significant changes in insulin and glucagon plasma levels were found in the SHR rats after palm^11^-PrRP31 administration. The treatment decreased the HOMA index in the SHROB but not the SHR rats (Table 1).

No significant changes in liver or kidney weights were observed, though the heart weight decreased in the SHR rats (Table 1). The urea concentration was not changed. The SBP did not change after 3 weeks of treatment in either rat (Table 1).

Treatment with the PrRP31 analog tended to decrease all circulating pro-inflammatory cytokine levels tested (IL-10, TNF-α, IL-6, and IL-1β) and increase anti-inflammatory IL-10 in both genotypes. The decrease in pro-inflammatory IL-1β in both SHR and SHROB rats and the decrease in pro-inflammatory IL-2 in the SHROB rats were both significant (Suppl. Figure 1).

The insulin signaling cascade was explored in the hypothalami by immunochemistry. PI3K was significantly increased in both phenotypes (Fig. 3b) and significant enhancement of IRβ was observed in the SHROB rats (Fig. 3c). A significant increase in MAPK/ERK1/2 phosphorylation occurred after the treatment in both SHR and SHROB rats (Fig. 3d).

Changes in the expression of several genes related to energy metabolism were also investigated. In the IPAT, palmitoylated PrRP31 analog treatment significantly increased Irs1 and Srebf1 mRNA expression in both genotypes, and Irs2 expression in the SHROB rats. In the SHROB rats, the treatment induced a significant decrease in liver Pparα mRNA expression (Table 2 and Suppl. Figure 2).

## Discussion

Koletsky rats (SHROB) are often used as a highly inbred animal model of metabolic syndrome. Though various other strains have been developed to study metabolic syndrome, SHROB is the only model for exploring all the mechanisms and interactions of obesity, hypertension, hyperlipidemia, and salt sensitivity^19^. Moreover, SHROB rats are extremely insulin-resistant because of their null mutation in the leptin receptor gene^15^. In our present study, the metabolic parameters of SHROB male rats and their age- and gender-matched SHR controls were determined at the age of 16 weeks (before the experiment) and 19 weeks (after the experiment).

In agreement with the literature data, we have demonstrated higher fat and BW, normal fasting glucose, but impaired glucose tolerance after an oral load, which worsened metabolic parameters and manifested as higher triglycerides, cholesterol, insulin, and leptin plasma levels in the SHROB rats compared to the lean SHR controls^15, 20, 21^. In contrast, we have observed lower fasting plasma FFA and glucagon levels in the SHROB rats compared with the SHR controls.

The reduced expression of the insulin receptor was linked to attenuated insulin signaling in fat^15, 21^. In our study, a decreased insulin receptor level in hypothalamus was observed in the SHROB compared to SHR rats. Additionally, the expression of several genes related to lipogenesis in adipose tissue and liver were significantly higher in the SHROB strain.

In agreement with published studies^22, 23^, negligible UCP-1 mRNA expression was detected in BAT from SHROB rats, pointing to a possibly distorted/attenuated energy expenditure in this strain.

In comparison with normotensive Wistar-Kyoto rats (results not shown), the SHROB rats are hypertensive, though SBP tended to be lower in comparison with lean SHR rats, which similar to the study by Friedman et al.^15^.

The main goal of this study was to examine whether our novel palmitoylated PrRP analog (palm^11^-PrRP31) could accomplish its potential anti-obesity and anti-diabetic effects in SHROB rats lacking leptin signaling and if its effect depends on functional leptin.

The most important result of this study is the marked improvement in glucose tolerance after palm^11^-PrRP31 treatment in both the SHROB and SHR rats; this result was similar to the observations in diet-induced obese Sprague Dawley rats^8^. Fasting normoglycemia was not altered by PrRP analog treatment, but the treatment significantly improved glucose tolerance in both genotypes. The improved glucose tolerance in the SHROB rats was accompanied by a significant decrease in plasma insulin levels and a subsequent decrease in the HOMA index and insulin/glucagon ratio.

The simultaneous decrease in insulin and increase in plasma glucagon levels resulted in a decrease in the insulin/glucagon ratio in the SHROB rats after palm^11^-PrRP treatment. As insulin blocks and glucagon stimulates the release of FFAs from adipocytes, the decreased insulin/glucagon ratio could be due to the increase in FFA levels registered in this study. The enhanced FFA levels were probably not related to the increased lipogenesis or the mRNA expression of related genes in the adipose tissue. Increased Irs1 and Irs2 mRNA expression in the IPAT and Irs1 expression in the SCAT could be a result of the decreased insulin levels. Decreased PPARα expression could also potentially enhance FFA levels. Similarly, hepatocyte-specific PPARα knockout mice had an increased FFA level^24^.

It is tempting to speculate that palm^11^-PrRP31 markedly improved liver insulin sensitivity with regard to decreased ectopic lipid storage in spite of the enhanced FFA levels. Recently, liver triglyceride production in type-2 diabetes was shown to be dependent on FFA levels rather than circulating insulin levels^25^.

Finally, palm^11^-PrRP31 treatment increased insulin receptor and PI3K levels in the hypothalami of both genotypes. However, the levels of the proteins mentioned do not correlate with activation that would specifically affect hypothalamic insulin signaling. Increased MAPK/ERK1/2 phosphorylation in the hypothalamus could be a result of either insulin or PrRP effects. For PrRP, ERK1/2 is its main activation pathway through its GPR10 receptor^26^.

It is accepted that leptin and insulin act together in the hypothalamus in order to target energy homeostasis^27^. Inhibition of food intake is mediated by both STAT3 through leptin receptor LepRb activation and PI3K through insulin receptor IR activation.

palm^11^- PrRP31 treatment increased both PI3K and ERK activation in the hypothalamus, both pathways known to be activated by insulin. Both PrRP and palm^11^- PrRP31 were shown to activate preferentially ERK signaling^9, 26^ PI3K activation by PrRP in RC4B/C pituitary cells—where GPR10 receptors are abundantly expressed—was reported by others^28^. However, we cannot distinguish if ERK and PI3K activation in the hypothalamus were results of direct action of palm^11^-PrRP31 on GRP10 or more efficient insulin signaling.

In SHROB rats, the insulin-resistant liver still produces triglycerides but does not attenuate gluconeogenesis. Induced expression of the leptin receptor in the hypothalamus attenuated hepatic expression of the gluconeogenesis genes glucose-6-phosphatase and phosphoenolpyruvate kinase^29^. The expression of the genes mentioned was not changed by palm^11^-PrRP31 treatment in the liver (not shown), meaning that palm^11^-PrRP31-induced ERK1/2 activation in the hypothalamus did not impact gluconeogenesis.

Thus, our novel palmitoylated PrRP31 analog was capable of ameliorating glucose tolerance and attenuating hyperinsulinemia and the insulin/glucagon ratio in SHROB rats similar to previously described treatments with angiotensin-converting enzyme inhibitors, angiotensin receptor 1 blockers^30–32^, a PPARγ agonist^33^, and the dipeptidyl peptidase IV inhibitor sitagliptin^20^, though most likely via a different mechanism of action and by targeting different receptor(s).

The three-week treatment of SHROB rats and their SHR littermates with our stable PrRP analog markedly decreased food intake and BW in the SHR but not in the SHROB rats. This result is in good agreement with our recent study on ZDF rats where 2-week treatment with the *N*-palmitoylated PrRP31 analog lowered food intake but did not decrease BW; this is in contrast to the effect of this analog in diet-induced obese Sprague Dawley rats^8^. We can speculate that intact leptin signaling is an important prerequisite for the PrRP BW-lowering effect. This explanation is further supported by our unpublished results on diabetic *db/db* mice with leptin receptor deficiency, where treatment with palmitoylated PrRP31 also had no effect on BW. However, glucose tolerance was markedly improved by this treatment in both the SHROB and SHR rats. This finding suggests that the positive effects of palm^11^-PrRP31 on glucose tolerance are mostly independent of its effect on BW, and makes this compound an interesting candidate not only for the treatment of obesity but also for the treatment of prediabetes/diabetes.

Adipose tissue is a secretory tissue that produces various substances that have specific functions in organisms^34^. Some of these substances are cytokines (e.g., IL-6 and TNF-α) whose production is known to be increased in obesity^35^ or hyperglycemia^36^, which suggests a higher prevalence of chronic inflammation in metabolic syndrome. However, in our study, higher cytokine production was not revealed in the obese SHROB rats compared with the lean SHR lean rats. In contrast, treatment with the palmitoylated PrRP31 analog tended to decrease pro-inflammatory cytokine levels, specifically promoting a significant decrease in IL-1β and a non-significant increase in anti-inflammatory cytokine IL-10 in both genotypes, which points to possible anti-inflammatory activities by palm^11^-PrRP31.

In conclusion, our study has shown that 3-week administration of lipidized PrRP analog markedly improved glucose tolerance in both lean SHR rats and SHROB rats despite only promoting a modest effect on BW in SHROB rats. Collectively, these findings suggest that improving glucose tolerance is mostly independent of anti-obesity effects, which make palm^11^-PrRP31 an interesting candidate to directly target prediabetes/diabetes along with obesity. Furthermore, as the effects of palm^11^-PrRP31 were observed in leptin receptor-deficient SHROB rats, improvements in glucose metabolism appear to be completely independent of leptin signaling. Taken together, our data suggest that functional leptin is required for the anorexigenic but not for the anti-diabetic effects of lipidized PrRP.

## Electronic supplementary material

Supplemental figures 1 and 2
