# Supplementary material for: Lipidized prolactin-releasing peptide improved glucose tolerance in metabolic syndrome: Koletsky and spontaneously hypertensive rat study
Source: Nutr Diabetes. 2018 Jan 16;8:5. doi: 10.1038/s41387-017-0015-8 (PMC5851428; doi:10.1038/s41387-017-0015-8)
Supplement: Supplementary file 1 — Supplemental figures 1 and 2 [file 41387_2017_15_MOESM1_ESM.docx]

**Supplemental Data**

**Supplemental Figure 1:** **Plasma levels of cytokines**.

Cytokines (interleukin (IL) 10 **(A)**, 1β **(B)**, tumor necrosis factor α (TNF-α) **(C)** and IL-10 **(D)**) were measured in fasted plasma after 21-day intraperitoneal treatment with palm^11^-PrPR31 at a dose of 5 mg/kg by MILIPLEX MAP rat cytokine bead panel (Millipore, St. Charles, MI, USA). Data are presented as means ± S.E.M. Statistical analysis was performed by unpaired t-test. Significance is *P<0.05, *vs* the vehicle-treated control group (n=8).

**
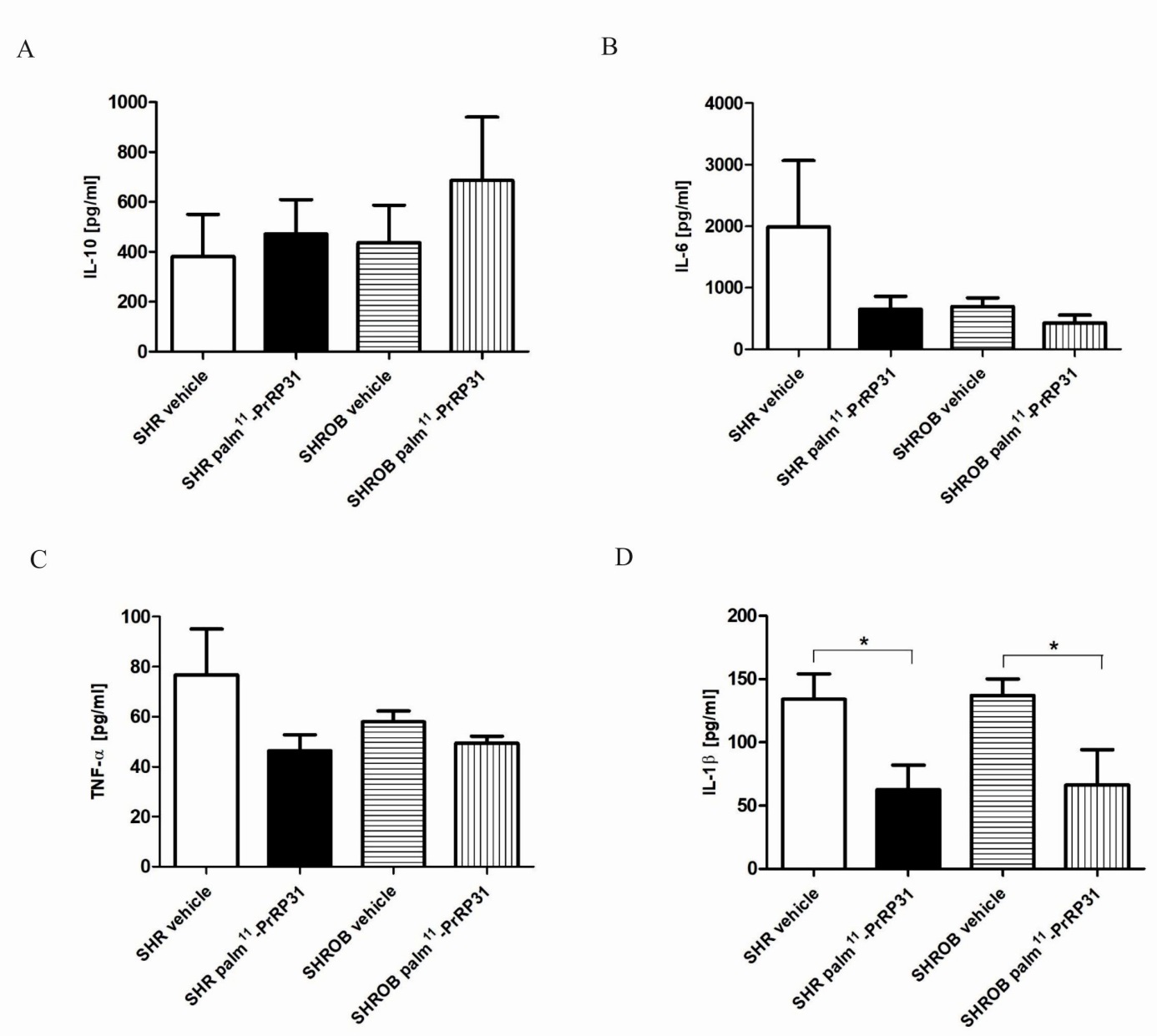
**

**Supplemental Figure 2: Chronic effect of palm^11^-PrRP31 on mRNA expressions** of Acaca, Lep, Lpl, Pparγ, Scd1 and Glut4 in the SCAT **(A)**, Srebp1, Irs1 and Irs2 in the IPAT **(B)**, Glut4 and UCP-1 in the BAT **(C)**, Acaca, Fasn, Pparα and Pparγ in the liver **(D)** of Koletsky rats. mRNA expressions were determined after the 21-day intraperitoneal treatment with palm^11^-PrPR31 at a dose of 5 mg/kg. Data are presented as mean ± S.E.M. The data were normalized to B2m and analyzed by unpaired t-test, significance is *P<0.05, *P<0.01, ***P<0.001 vs the respective control group (n=8).


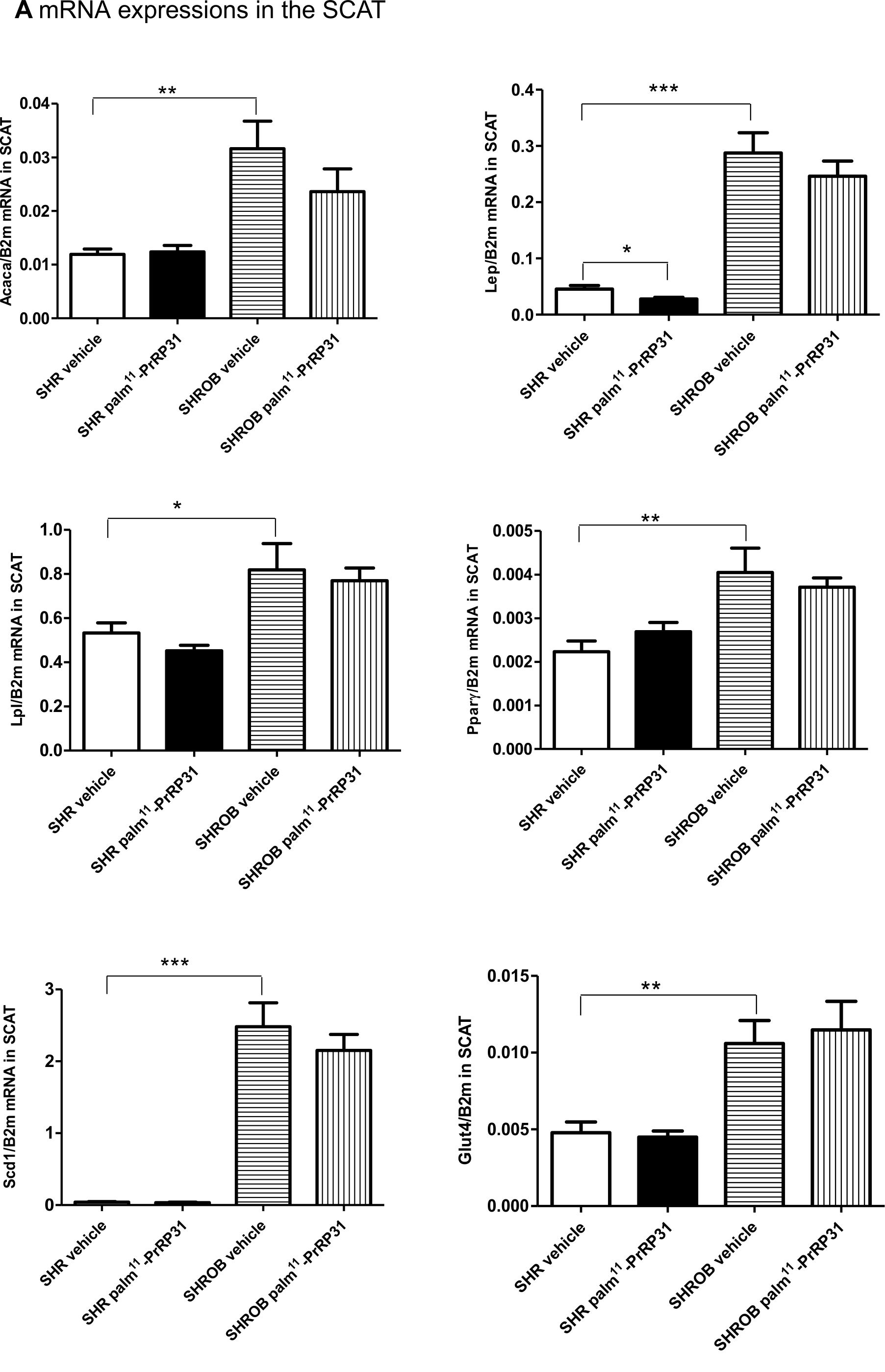


**
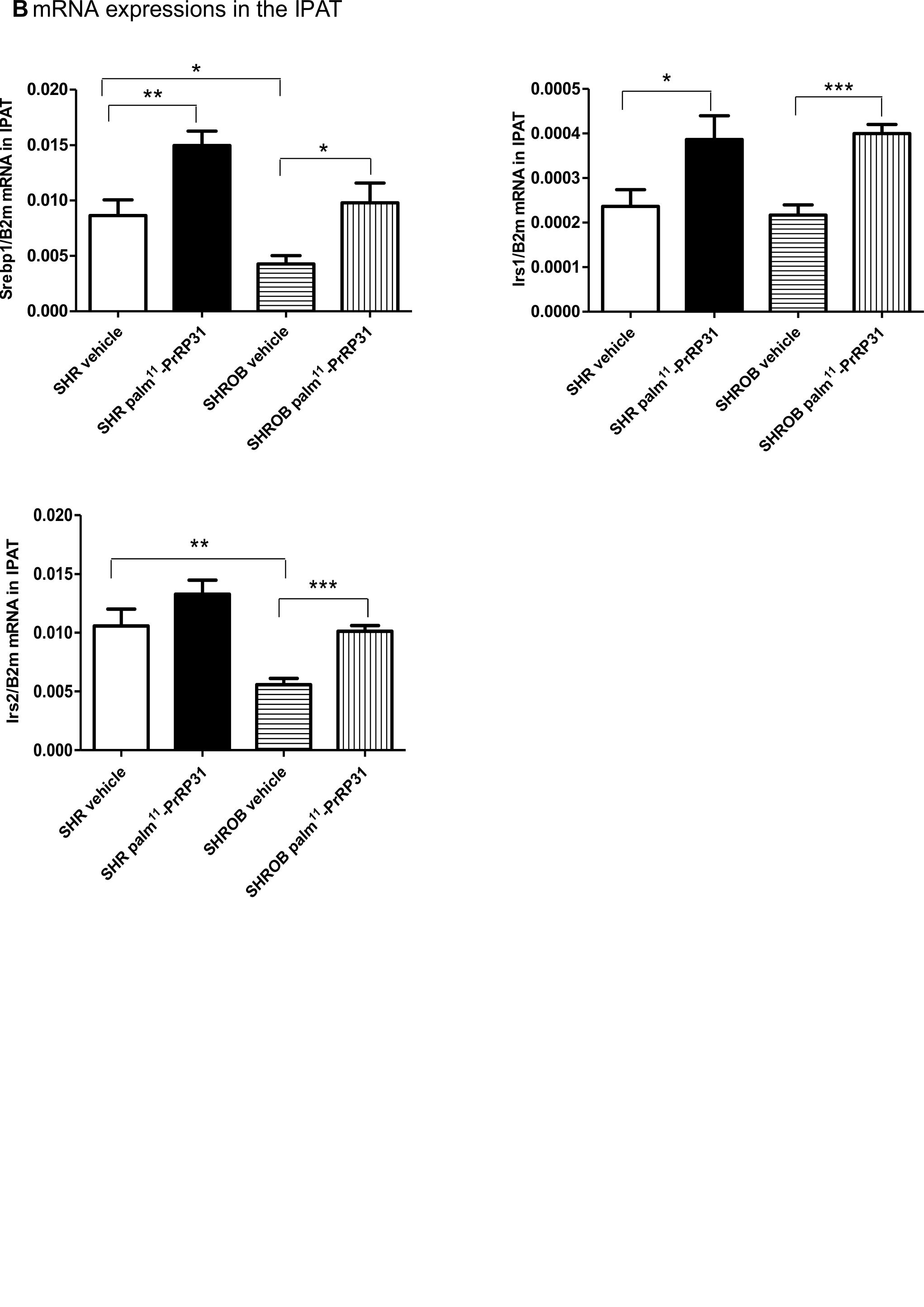
**

**
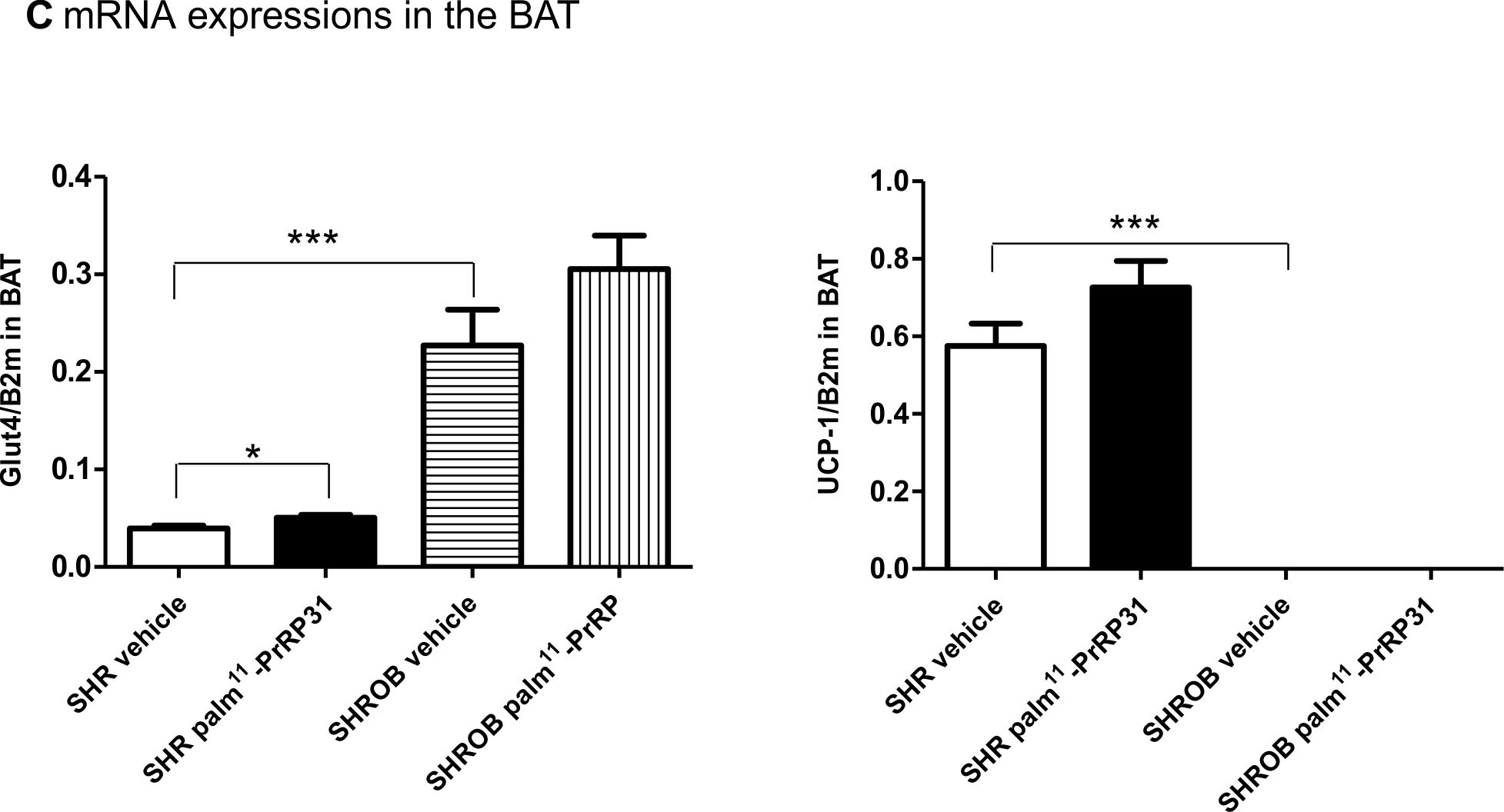
**

**
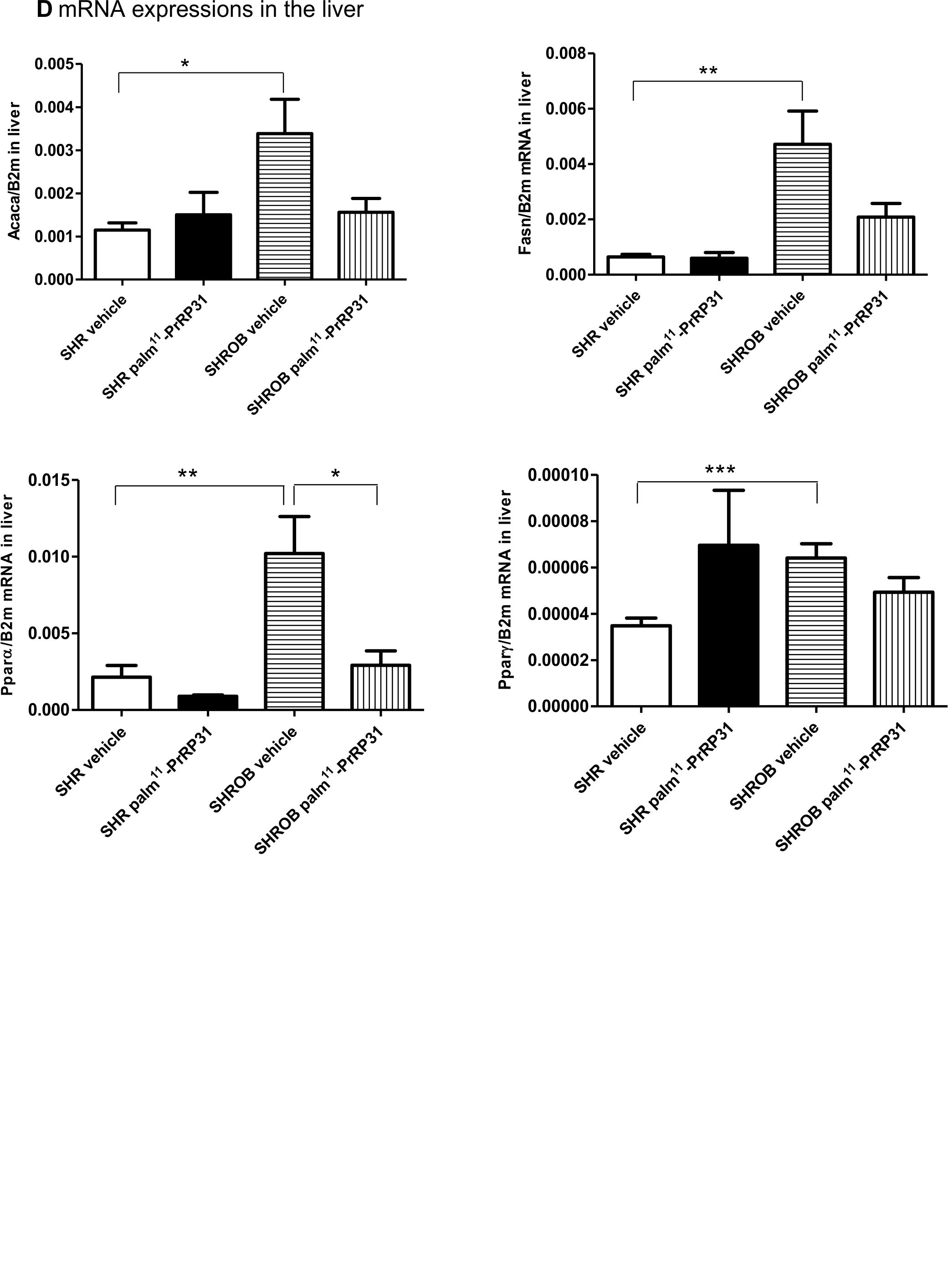
**
